# Cupping for neurodermatitis: A protocol of systematic review and meta-analysis

**DOI:** 10.1097/MD.0000000000022586

**Published:** 2020-10-02

**Authors:** Li Peng, Qianying Yu, Jie Zhang, Xiongfei Mi, Wenxia Lin, Yuesi Qin, Ying He, Jing Guo, Min Xiao, Mingling Chen

**Affiliations:** a Hospital of Chengdu University of Traditional Chinese Medicine; b Chengdu Integrated TCM & Western Medicine Hospital, Chengdu, Sichuan Province, P. R. China.

**Keywords:** cupping therapy, neurodermatitis, protocol, systematic review

## Abstract

**Background:**

Neurodermatitis is a common inflammatory and allergic disease, characterized by itching and lichenification plaque. Some studies have reported cupping therapy (CT) for the treatment of neurodermatitis. However, the effectiveness and safety are still uncertain. This study aims to evaluate the efficacy and safety of CT for the treatment of patients with neurodermatitis.

**Methods:**

We will retrieve the following electronic databases systematically: Pubmed, Web of Science, Embase, the Cochrane Library, Chinese Scientific Journal Database, China National Knowledge Infrastructure Database, Chinese Biomedical Literature Database, and Wanfang database from their inception to December 2020. Other literature resources will be manually searched. Published randomized controlled trials (RCTs) and quasi-randomized controlled trials (q-RCTs) on the topic will be retrieved by 2 investigators independently. We will apply a fixed-effect model or random effect model basis on the heterogeneity test and employ with RevMan 5.3 software for data synthesis. The total clinical effective rate will be selected as the primary outcome, skin disease quality of life index score, recurrence rate, and adverse events as secondary outcomes.

**Results:**

This study will comprehensively summarize the high-quality trials to determine the efficacy and safety of CT for the treatment of patients with neurodermatitis.

**Conclusion:**

Our systematic review will present evidence for the efficacy and safety of CT to neurodermatitis patients.

**OSF Registration number:**

DOI 10.17605/OSF.IO/6DCM3.

## 1 Introduction

Neurodermatitis is a common clinical chronic inflammatory and allergic skin disease,[^1^,^2^] with the characteristic of a repeated itch-scratch cycle. Neurodermatitis divides into disseminated or localized skin lesions according to the extent of skin lesions. The clinical symptoms cover dry, lichenoid (caused by excessive scratching),^[3]^ thickened skin lesions, accompanied by hyperpigmented. Lichen simplex chronicus (LSC) also acts as a common form of neurodermatitis.^[4]^ Skin lesions are frequently found at the neck (sides), ankles, scalp, vulva, extensor forearms, pubis, scrotum, and perianal.^[5]^ Neurodermatitis affects females more than males, among all ages in the population^[3]^ whether adults or children.^[6]^ Certain types of neurodermatitis can be divided into primary and secondary dermatitis (eg, psoriasis).^[6]^ The psychogenic factors, hot water, rubbing, and scratching will induce or aggravate the condition. It was reported that compulsive habits, anxiety, venereophobia,^[5]^ depression, and dissociative experiences contribute to the disease.^[7]^ Patients with neurodermatitis are associated with psychosocial burden, poor flexibility, worse social relationship, sleep disturbance, and sexual dysfunction,[^8^,^9^] low quality of life.^[10]^

Management of neurodermatitis faces tough challenges. Topical corticosteroids, immunomodulators (Tacrolimus, Pimecrolimus), antipruritics (Doxepin, Capsaicin, Aspirin), antiepileptics, antihistamines, antidepressants are recommended to control the condition.^[11]^ All of these therapies may alleviate the symptom with the short term, but the considerable side effects include erythema, hyperpigmentation, burning, and dryness limit the treatment.^[11]^ Neurodermatitis tends to recur, due to the difficulties of impeding the itch-scratch cycle. To some extent, emotion-regulating skills play the therapeutic roles to the patients with psychological abnormality.^[3]^ As reported, lifestyle improvements such as silk fabric underwear for vulvar LSC,^[6]^ avoiding exposure to allergens, mindfulness-based cognitive hypnotherapy,^[12]^ and homeopathy^[13]^ can also alleviate the condition.

Based on the occurrence of side effects caused by conventional medication for prolonged employment,^[13]^ dermatologists and patients show solicitude for multifaceted effective methods to control the chronic and relapsing nature of neurodermatitis.^[6]^ Acupuncture is most commonly examined as an alternative treatment for dermatitis, urticaria, and pruritus.^[14]^ It is well known that cupping therapy (CT) is one of the effective and significant methods of acupuncture in the history of China and Egypt.^[15]^ CT includes several forms such as dry cupping, wet cupping, and others.^[16]^ What's more, CT relieved the symptoms, and relapse of neurodermatitis has been reported for a long time.[^17^,^18^] Not only that, but CT also obtained satisfactory results involving fibromyalgia syndrome,^[19]^ migraine headache,^[20]^ psoriasis Vulgaris,^[21]^ chronic urticaria,^[22]^ and others. Due to no systematic reviews concerning this topic, this study will evaluate the effectiveness and safety of CT for neurodermatitis according to the authoritative Cochrane recommendations.

## 2 Methods

### 2.1 Study registration

This study of systematic review and meta-analysis was registered on the open science framework with the Registration number of DOI 10.17605/OSF.IO/6DCM3. Our study will be implemented based on the Cochrane Handbook and the Preferred Reporting Items for Systematic Reviews and Meta-Analyses Protocols statement guidelines.^[23]^

### 2.2 Ethics

The data of our study will be obtained from published literature, so ethical approval will be not required.

### 2.3 Eligibility criteria for study inclusion

#### 2.3.1 Study types

This study will collect data from published randomized controlled trials (RCTs) and quasi-randomized controlled trials (q-RCTs) on CT for the patients in neurodermatitis, which were reported in English and Chinese. Other researches, such as nonclinical trials, non-RCTs, reviews, case series, animal, and cell studies will be excluded.

#### 2.3.2 Types of participants

All the sufferers in our study have been diagnosed as neurodermatitis in line with the authoritative diagnostic criteria.

This study will not stipulate the age, sex, race, belief, nationality, occupation, mental states such as anxiety, depression, and disease course, degree, number of recurrences, degree of itching, thickness and location of skin lesions, and inducing or aggravating factors of any participants.

#### 2.3.3 Type of interventions

Interventions of CT will be included as the experimental group, with no restrictions on the methods of CT, such as dry cupping, wet cupping, moving cupping, flash cupping, and no restrictions on the materials of the cans, such as glass, plastic, and bamboo cans, and so on. This study will also include 2 cupping treatment operations and cupping combines with other active treatment methods. In addition to CT, the intervention measures of the control group should be the same as the experimental group.

### 2.4 Types of outcome measures

The primary outcome of this study will be the total clinical effective rate, which will be judged by the calculation of the Eczema Area and Severity Index (EASI) or Visual Analogue Scale (VAS) scores. Several secondary outcomes indicators will also be defined, including skin disease quality of life index score, recurrence rate, and adverse events of the therapy.

### 2.5 Search strategy for study conducting

#### 2.5.1 Electronic searches

Two investigators (LP and QY) will execute the structured and systemic literature retrieval without interfering with each other in the following 10 electronic bibliographic databases: Pubmed, Web of Science, Embase, the Cochrane Library, Chinese Scientific Journal Database, China National Knowledge Infrastructure Database, Chinese Biomedical Literature Database, and Wanfang database. The search period will be from the establishment of the database to December 2020 in our study.

This research will introduce the following search glossary to filter out literature that conforms with the criterion: neurodermatitis, LSC, lichen simplex, dermatoneurosis, neurodermatosis, neurodermatitides, niu pixuan, she lingchuang, CT, cupping, CT, wet cupping, dry cupping, pricking cupping, moving cupping, flash cupping, bloodletting, pricking blood therapy. Table 1 showed an example of the search strategy for PubMed. We will appropriately adjust the search strategy according to the different databases.

**Table 1:**
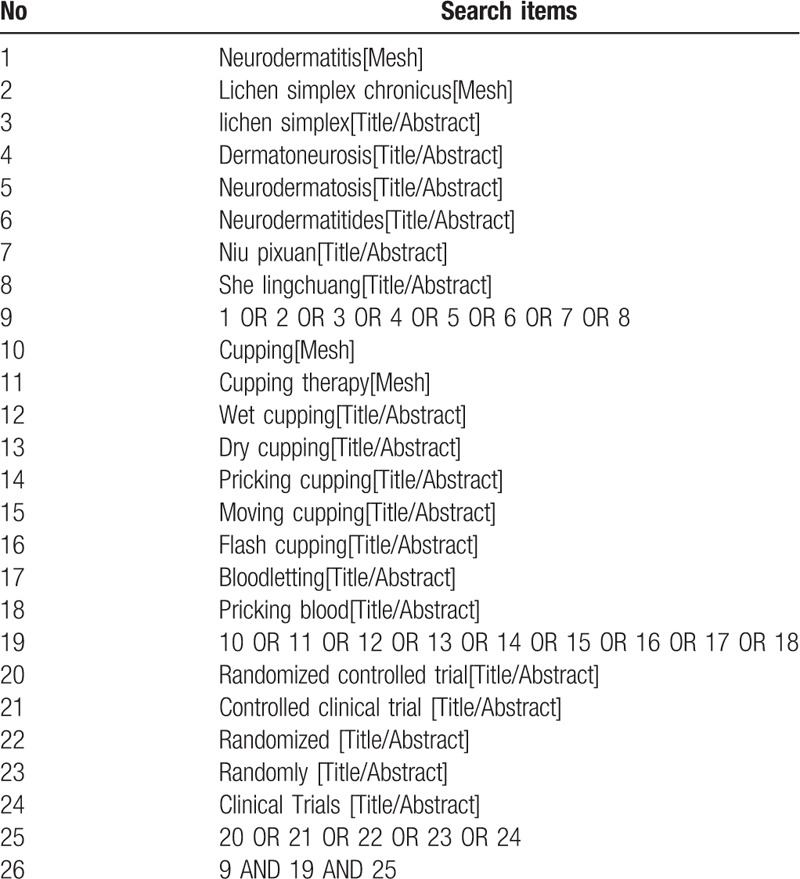
The search strategy applied in PubMed.

#### 2.5.2 Other resources

The other literature resources concern of CT for neurodermatitis will also be browsed, such as meta-analysis, RCTs, books, conference articles, registers of clinical trials to find potential information.

### 2.6 Data collection and analysis

#### 2.6.1 Selection of studies

**Figure 1:**
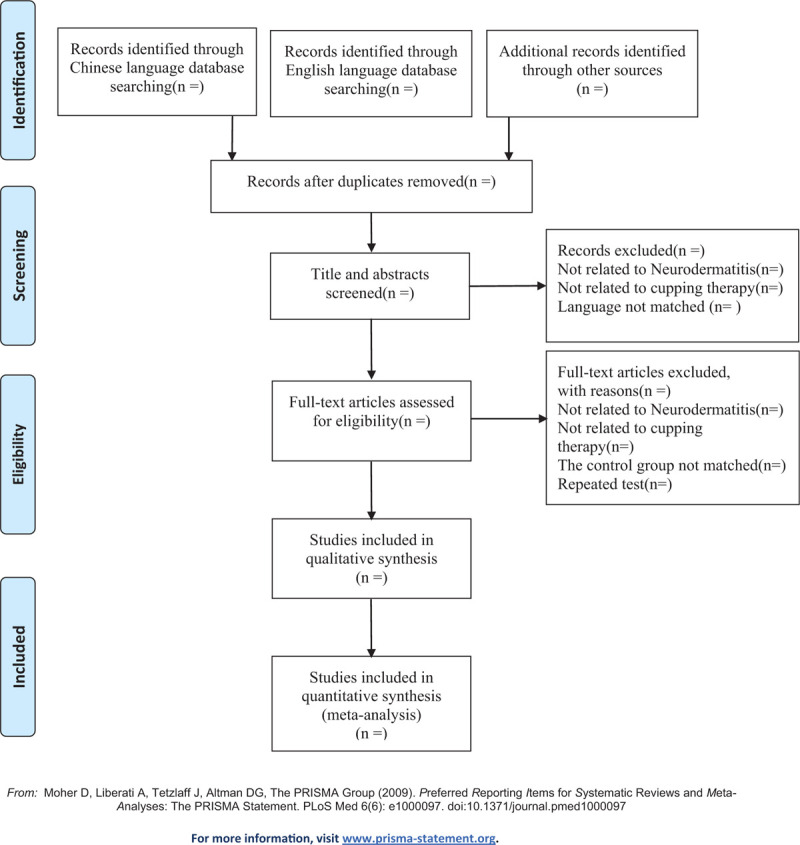
Flow diagram of the study selection process.

All the files from the literature database will be brought in EndnoteX9 software for management and deleting duplicate contents after all researchers comprehended the relevant rules. To screen qualified trials, two researchers (LP and QY) will browse the titles, abstracts, keywords from the selected reports following the regulations strictly and exclude irrelevant researches. Furthermore, the full-text of articles should be preserved and archived for further investigation. The Excel data will be applied for the recording of exclusion details. Another author (JZ) will act as a referee to resolve any divergence in the course of research. Figure 1 displayed the flow diagram of our study in detail.

#### 2.6.2 Data extraction and management

The qualifications of included studies will be independently censored by 2 authors (WX and YS), then who will collect data and import it into the software. The extracted data will include general information, participant characteristics, inclusion and exclusion criteria, sample size, randomization, blinding methods, research methods, type of cupping interventions, control group, outcome, results, adverse events, conclusions, recurrence conditions, and other controversial details. In the progress of data collecting, the consultation of 2 authors or the opinion of the third author (JZ) will be accepted to the discrepancy.

#### 2.6.3 Missing data management

When we find blurry or inadequate details in our included trials, we will send emails or make telephone calls to the original authors for more information. If not feasible, the available data and possible effects of lost information will be analyzed based on the included trials.

#### 2.6.4 Risk of bias assessment

Cochrane Risk of Bias Tool will be applied to inspect the risk of bias of selected trials by 2 independent reviewers (JG and MX). Our study mainly focuses on 7 aspects of each trial, such as random sequence generation, allocation sequence concealment, blinding of participants and personnel, and so on. The bias of results covers as low risk, high risk, and uncertain risk. Differences will be resolved by discussion with the third author until consensus be achieved.

#### 2.6.5 Treatment effect measurement

The treatment effect will be measured by mean differences (MD) or standardized MD together with 95% confidence intervals (CIs) for the continuous outcomes. And for the dichotomous outcome, risk ratio or odds ratio together with 95% CIs will be available.

#### 2.6.6 Data analysis

The data analysis will be employed with RevMan 5.3 software from the Cochrane Collaboration.

And in our study, the level of heterogeneity will be quantified by the *I* ^2^ statistic. The *I* ^2^ value <50%, demonstrating the studies are homogeneous or low heterogeneity, with a fixed-effect model forthputting. In contrast, *I* ^2^ value ≥50%, demonstrating the studies are substantial heterogeneity, with a random-effect model forthputting. Then, possible reasons for heterogeneity will be executed of sensitivity analysis or subgroup analysis. Descriptive analysis of results will be performed when considerable heterogeneity causes the analysis infeasible.

#### 2.6.7 Subgroup analysis

Consideration of the possible impact of cupping types, intervention methods, and outcome measurements to the conspicuous heterogeneity, subgroup analysis will be conducted.

#### 2.6.8 Sensitivity analysis

The robustness and reliability of the review outcome will be judged by sensitivity analysis in the statistical model, sample size, missing data, and others.

#### 2.6.9 Reporting bias assessment

When we select >10 studies consistent with conditions, a funnel plot and Egger regression test will be performed to appraise the reporting biases.

#### 2.6.10 Evidence quality

The quality of evidence will be ranked as 4 levels: high, moderate, low, and very low according to the Grading of Recommendations Assessment, Development and Evaluation (Version 3.6, The grading of recommendations assessment, development, and evaluation Working Group) instrument for included studies.^[24]^

## 3 Discussion

Neurodermatitis exerts a negative influence on the quality of life and skin health, because of the scratching caused by unbearable and repeated itching. Lichenification of the skin can also affect the appearance, especially the bare parts of women. What's worse, the management of the disease may consume public health care resources.^[25]^ The effective methods support the urgent need for neurodermatitis should be found. As the significant alternative treatment for dermatitis among the world medical practice, the effectiveness and safety of CT for neurodermatitis should be summarized based on the current studies. Thus our study may provide objective evidence for the clinicians to perform the preferred strategy.

## Author contributions

**Conceptualization:** Li Peng.

**Data curation:** Li Peng, Qianying Yu, Jie Zhang, Wenxia Lin.

**Formal analysis:** Li Peng, Qianying Yu.

**Funding acquisition:** Jing Guo.

**Investigation:** Yuesi Qin, Ying He, Jing Guo, Min Xiao.

**Methodology:** Li Peng, Qianying Yu, Jie Zhang.

**Supervision:** Xiongfei Mi, Jing Guo, Min Xiao, Mingling Chen.

**Writing – original draft:** Li Peng.

**Writing – review & editing:** Mingling Chen, Min Xiao, Jing Guo.

## References

[1] Feng X Yang M Yang Z Abnormal expression of the co-stimulatory molecule B7-H3 in lichen simplex chronicus is associated with expansion of Langerhans cells. Clin Exp Dermatol 2020;45:30–5. 31056761 10.1111/ced.14001

[2] Santos-Hövener C Kuntz B Frank L Zur gesundheitlichen Lage von Kindern und Jugendlichen mit Migrationshintergrund in Deutschland: Ergebnisse aus KiGGS Welle 2 [The health status of children and adolescents with migration background in Germany: Results from KiGGS Wave 2]. Bundesgesundheitsblatt Gesundheitsforschung Gesundheitsschutz 2019;62:1253–62. 31529190 10.1007/s00103-019-03012-x

[3] Lotti T Buggiani G Prignano F Prurigo nodularis and lichen simplex chronicus. Dermatol Ther 2008;21:42–6. 18318884 10.1111/j.1529-8019.2008.00168.x

[4] Charifa A Badri T Harris BW Lichen Simplex Chronicus In: StatPearls. Treasure Island (FL): StatPearls Publishing; 2020. 29763167

[5] Agulló-Pérez AD Hervella-Garcés M Oscoz-Jaime S Perianal dermatitis. Dermatitis 2017;28:270–5. 28338543 10.1097/DER.0000000000000274

[6] Corazza M Borghi A Minghetti S Effectiveness of silk fabric underwear as an adjuvant tool in the management of vulvar lichen simplex chronicus: results of a double-blind randomized controlled trial. Menopause 2015;22:850–6. 25608275 10.1097/GME.0000000000000410

[7] Konuk N Koca R Atik L Psychopathology, depression and dissociative experiences in patients with lichen simplex chronicus. Gen Hosp Psychiatry 2007;29:232–5. 17484940 10.1016/j.genhosppsych.2007.01.006

[8] Ermertcan AT Gencoglan G Temeltas G Sexual dysfunction in female patients with neurodermatitis. J Androl 2011;32:165–9. 20864649 10.2164/jandrol.110.010959

[9] Juan CK Chen HJ Shen JL Lichen simplex chronicus associated with erectile dysfunction: a population-based retrospective cohort study. PLoS One 2015;10:e0128869. 26076496 10.1371/journal.pone.0128869 PMC4468076

[10] An JG Liu YT Xiao SX Quality of life of patients with neurodermatitis. Int J Med Sci 2013;10:593–8. 23533146 10.7150/ijms.5624 PMC3607245

[11] Juarez MC Kwatra SG A systematic review of evidence based treatments for lichen simplex chronicus [published online ahead of print, 2020 Mar 6]. J Dermatolog Treat 2020 1–9. 10.1080/09546634.2019.1708856 31884840

[12] Shenefelt PD Mindfulness-based cognitive hypnotherapy and skin disorders. Am J Clin Hypn 2018;61:34–44. 29771216 10.1080/00029157.2017.1419457

[13] Gründling C Schimetta W Frass M Real-life effect of classical homeopathy in the treatment of allergies: a multicenter prospective observational study. Wien Klin Wochenschr 2012;124:11–7. 22138796 10.1007/s00508-011-0104-y

[14] Ma C Sivamani RK Acupuncture as a treatment modality in dermatology: a systematic review. J Altern Complement Med 2015;21:520–9. 26115180 10.1089/acm.2014.0274

[15] Qureshi NA Ali GI Abushanab TS History of cupping (Hijama): a narrative review of literature. J Integr Med 2017;15:172–81. 28494847 10.1016/S2095-4964(17)60339-X

[16] Soliman Y Hamed N Khachemoune A Cupping in dermatology: a critical review and update. Acta Dermatovenerol Alp Pannonica Adriat 2018;27:103–7. 29945267

[17] Zhang Y Zhou JW Huang S Observation on the therapeutic effect of a red-hot needle therapy combined with blood-letting puncture and cupping for treatment of neurodermatitis [in Chinese]. Chin Acup Moxib 2007;27:252–4. 17585666

[18] Jiang QQ Cai CX Cong YY A study on the improvement of pruritus in neurodermatitis by combination of fire needle and cupping therapy [in Chinese]. Chin J Derma Integr Tradit West Med 2018;17:418–20.

[19] Lauche R Spitzer J Schwahn B Efficacy of cupping therapy in patients with the fibromyalgia syndrome—a randomised placebo controlled trial. Sci Rep 2016;6:37316. 27853272 10.1038/srep37316 PMC5112514

[20] Ersoy S Benli AR Continue or stop applying wet cupping therapy (al-hijamah) in migraine headache: a randomized controlled trial. Complement Ther Clin Pract 2020;38:101065. 31668556 10.1016/j.ctcp.2019.101065

[21] He B Lin ZF Clinical efficacy of moving cupping therapy for treatment of psoriasis vulgaris with blood stasis syndrome and its effect on expression of serum tumor necrosis factor alpha and vascular endothelial growth factor [in Chinese]. J Guangzhou Univ Tradit Chin Med 2020;37:64–8.

[22] Wang Y Xiao JH Li D Clinical study of chronic urticaria by acupuncture combined with pricking-cupping [in Chinese]. JCAM 2017;33:12–4.

[23] Moher D Shamseer L Clarke M Preferred reporting items for systematic review and meta-analysis protocols (PRISMA-P) 2015 statement. Syst Rev 2015;4:1. 25554246 10.1186/2046-4053-4-1 PMC4320440

[24] Zhang J Yu Q Peng L Cupping for psoriasis vulgaris: a protocol of systematic review and meta-analysis. Medicine (Baltimore) 2020;99:e20348. 32443388 10.1097/MD.0000000000020348 PMC7253533

[25] Singam V Patel KR Silverberg JI Association of prurigo nodularis and lichen simplex chronicus with hospitalization for mental health disorders in US adults. Arch Dermatol Res 2020;312:587–93. 32078024 10.1007/s00403-020-02046-5

